# Notch Signaling in Health and Disease

**DOI:** 10.3390/ijms242216113

**Published:** 2023-11-09

**Authors:** Paola Maura Tricarico, Sergio Crovella

**Affiliations:** 1 Institute for Maternal and Child Health—IRCCS “Burlo Garofolo”, Department of Advanced Diagnostics, 34137 Trieste, Italy; 2 Laboratory of Animal Research Center (LARC), Qatar University, Doha P.O. Box 2713, Qatar; sgrovella@qu.du.qa

The Notch signaling pathway, a vital and evolutionarily conserved regulator of cellular processes, intricately shapes both health and disease. In the realm of health, Notch signaling assumes a pivotal role in the skin homeostasis, orchestrating primary growth arrest and progressive keratinocyte differentiation processes [1]. Moreover, Notch signaling is also critical in other adult organs and tissues such as the liver, intestine, hematopoietic system, and skeletal muscle.

Its involvement in immune cell homeostasis and organ-specific immune responses accentuates its overarching significance in overall immune function [2]. Moreover, the pathway contributes to the integrity of the vasculature, the development of smooth muscle cells, and intricate processes such as osteogenic differentiation, highlighting its versatile role in maintaining physiological balance.

Conversely, dysregulation of Notch signaling is implicated in an array of diseases. In cancer, aberrant Notch activation is intricately linked to tumor initiation, progression, and chemoresistance across various malignancies [3]. Cardiovascular disorders, exemplified by myocardial infarction, showcase the cardioprotective effects of Notch signaling [4], while neurological conditions like Cerebral Autosomal Dominant Arteriopathy with Subcortical Infarcts and Leukoencephalopathy (CADASIL) underscore its role in hereditary strokes [5]. In essence, unraveling the nuanced dynamics of Notch signaling is paramount for understanding the molecular underpinnings of diseases and harnessing its therapeutic potential. The multifaceted involvement of Notch signaling in health and disease underscores its intricate nature, positioning it as a promising avenue for targeted interventions and personalized medicine.

This Special Issue titled “Notch Signaling in Health and Disease” delves into the diverse roles of the Notch signaling pathway in various physiological and pathological contexts. The manuscripts included in this compilation shed light on the intricate involvement of Notch signaling in myocardial infarction, immune responses, oral squamous cell carcinoma, CADASIL, vascular smooth muscle cells, osteogenic differentiation, renal vascular response, ovarian cancer, colon adenocarcinoma, glioblastoma, and epithelial ovarian cancer.

The review “Myocardial Infarction and Cardio protection” emphasizes the cardioprotective effects of Notch signaling in myocardial infarction, elucidating its role in reducing oxidative stress, preventing apoptosis, regulating inflammation, containing fibrosis, facilitating tissue revascularization, and orchestrating the proliferation and differentiation of cardiomyocytes. Notch signaling emerges as a potential therapeutic target for enhancing cardiac recovery post-myocardial infarction.

The review “Notch Signaling in Immune Responses and Sepsis” explores the dual role of Notch signaling in immune cell differentiation and its impact on immune signals. Focusing on conditions like sepsis, it navigates through the modulation of organ-specific immune responses. This review raises prospects for manipulating the Notch signaling pathway as a future therapeutic strategy for inflammatory diseases.

The research article “Notch Signaling in Oral Squamous Cell Carcinoma (OSCC)”, investigates the inhibition of Notch signaling in OSCC. The authors reveal its crucial role in promoting cell growth, cell cycle progression, migration, and invasion. The findings suggest the potential use of Notch signaling inhibitors as adjuvant treatments in OSCC patients.

“CADASIL and Blood Biomarkers” is focused on CADASIL, a hereditary stroke disorder, which is associated with mutations in the NOTCH3 gene. In this research article, authors introduce blood biomarkers related to the Notch signaling pathway, providing a basis for further research on their clinical utility in CADASIL and related diseases.

Focusing on the stability of CSL proteins that is central to Notch signal transduction, “Regulation of Notch Components” revealed conserved mechanisms of CSL protein turnover across species. The findings suggest that turnover of CSL proteins may regulate Notch signaling output at the transcriptional level.

Examining the indirect immobilization of Jagged-1 and its effects on osteogenic differentiation, the research article “Jagged-1 in Osteogenic Differentiation” delves into the proteomic profiles of dental pulp stem cells. The study suggests that Jagged-1 induces osteogenic differentiation through the regulation of proteins in the membrane compartment.

The research article “Renal Vascular Response and Notch3” investigates Notch3’s role in renal vascular response. The authors identified its essential role in excitation–contraction coupling and vascular-tone regulation in the kidney. Notch3 deficiency led to altered vascular reactivity, emphasizing its significance in renal physiology.

“Diagnostic Biomarkers in Ovarian Cancer” explored the dysregulation of the Notch pathway in ovarian cancer. This pilot study identified a panel of genes, including ARID1A, CTNNB1, FBXW7, and PPP2R1A, as potential diagnostic biomarkers for ovarian cancer.

“Notch4 in Colon Adenocarcinoma” investigated the clinical application of Notch4 in colon adenocarcinoma. This research article highlights its role as a potential prognostic marker in patients with this malignancy.

The research article “Combating Chemoresistance in Ovarian Cancer” addressed chemoresistance in ovarian cancer. The authors revealed that the inhibition of Notch signaling, in combination with resveratrol, can reverse the transformation of mesenchymal phenotypes to epithelial-like phenotypes, which affect invasion and stemness.

“CD109 in Epithelial Ovarian Cancer” unraveled the role of CD109 in epithelial ovarian cancer. and uncovered its positive correlation with drug resistance. The study suggests that CD109 induces drug resistance through the STAT3-NOTCH1 signaling axis.

This Special Issue provides a comprehensive overview of the diverse facets of Notch signaling, from cardiovascular health to cancer biology [6] emphasizing its potential as a therapeutic target in various diseases [7]. The insights derived from these manuscripts contribute to our understanding of the intricate roles played by Notch signaling in health and disease, paving the way for future research and clinical applications.
